# The complete chloroplast genome of *Lithocarpus hancei* (Benth.) Rehd (Fagaceae) from Zhejiang, China

**DOI:** 10.1080/23802359.2021.1935357

**Published:** 2021-06-21

**Authors:** Chen-Xin Ma, Hai-Fei Yan, Xue-Jun Ge

**Affiliations:** a Key Laboratory of Plant Resources Conservation and Sustainable Utilization, South China Botanical Garden, Chinese Academy of Sciences, Guangzhou, China; b University of Chinese Academy of Sciences, Beijing, China

**Keywords:** Fagaceae, *Lithocarpus hancei*, Phylogeny, plastid genome

## Abstract

*Lithocarpus hancei* (Benth.) Rehd is a widely distributed evergreen tree with broad-leaves that dominates the lower stories of the forest in China. Here, we sequenced and assembled the complete chloroplast genome of *L. hancei*. The genome is 161,304 bp with one large single copy (LSC: 90,585 bp), one small single copy (SSC: 18,959 bp), and two inverted repeat (IR) regions (IRa and IRb, each 25,880 bp). It contains 117 genes, including 80 protein-coding genes, 33 tRNA genes, and four rRNA genes. Phylogenetic analysis of 21 representative cp genomes of the Fagaceae suggests *Lithocarpus* is monophyletic with strong bootstrap support and also that *L. hancei* is closely related to *L. polystachyus*. The cp genome is important for constructing a robust phylogeny of *Lithocarpus* and Fagaceae for future study.

*Lithocarpus* Blume is a large genus classified in the Fagaceae with more than 300 species that is predominantly distributed in Asia (Huang and Bruce Bartholo 1999). China hosts 122 *Lithocarpus* species (Huang and Bruce Bartholo 1999). This genus plays an essential role in the evergreen broad-leaved forest (Huang and Bruce Bartholo 1999; Cannon and Manos 2003). *Lithocarpus hancei* (Benth.) Rehd 1919 is a widely distributed evergreen tree with broad-leaves that occurs under 2600 m in central and south China (Huang and Bruce Bartholo 1999). It is a shade-tolerant canopy species that lives in the shaded-story or canopy gaps and sometimes is codominant the lower stories with *Castanopsis* Spach and *Fagus* L. (Cao 2001). Chloroplast DNA (cp DNA) has been widely used for species identification, phylogeny reconstruction, demographic history tracing and species divergence studies (Yang et al. 2016; Yang et al. 2017; Alzahrani et al. 2020), because of its conserved genome structure and uniparental inheritance (Birky et al. 1983). However, only three cp genomes of *Lithocarpus* are available in GenBank, of which only the cp genome of *Lithocarpus balansae* (Drake) A.Camus has been fully annotated. To better understand the phylogenetic position of *L. hancei*, in this study we sequenced and assembled its chloroplast genome.

The voucher specimen (Li Buhang et al. BSZ142) was collected from Baishanzu Mountain, Zhejiang, China (27°32'25′'N, 118°57'49′'E) and deposited at the herbarium of South China Botanical Garden (SCBG, Feiyan Zeng, zengfeiy@scbg.ac.cn). Genomic DNA was extracted from silica gel-dried leaves using the CTAB method (Doyle 1987), and sequenced by the Illumina Hiseq X platform at the Beijing Genomics Institute (Wuhan, China). The clean reads were assembled using the program GetOrganelle v1.7.1 (Jin et al. 2020). The annotation was performed on DOGMA (Wyman et al. 2004) and manually corrected using Geneious 7.1.4 (Biomatters, Ltd, Auckland, New Zealand). The complete cp genome of *L. hancei* and 20 related species classified in the Fagaceae, plus two outgroups (*Corylus heterophylla* Fisch and *C. fargesii* Schneid) were aligned using MAFFT with auto alignment strategy (Katoh and Standley 2013). Phylogenetic analysis was performed using RAxML v8.2.12 with 1000 bootstrap replicates under the GTA + GAMMA model (Stamatakis 2014).

The complete cp genome of *L. hancei* is 161,304 bp in length with a GC content of 36.7%. The quadripartite structure of the cp genome has one LSC region of 90,585 bp, one SSC region of 18,959 bp, and a pair identical IR regions of 25,880 bp. A total of 117 genes were annotated including 80 protein-coding, 33 tRNA and four rRNA genes. Comparison of the *L. hancei* cp genome with the publicly available *L. balansae* genome using the program mVISTA (Mayor et al. 2000) under the LAGAN mode, indicated that the two were highly conserved, containing 80 protein-coding genes and four rRNA. However, *L. hancei* has 31 tRNA, while *L. balansae* contains 33 of these genes. The ML tree (Figure 1) fully supported a monophyletic *Lithocarpus* with *L. hancei* closely related to *L. polystachyus* Rehd. The cp genome of *L. hancei* will help us better construct a robust phylogeny of *Lithocarpus* and the Fagaceae in the future study.

**Figure 1.**
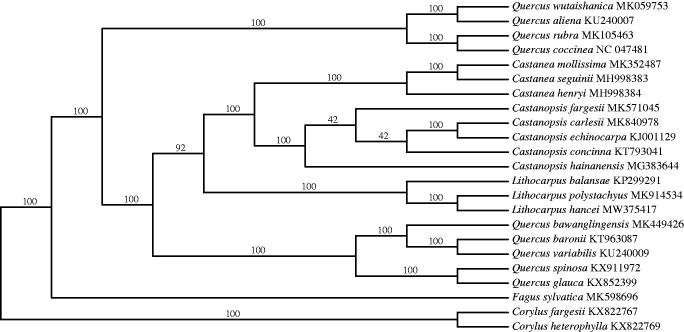
Phylogenetic analysis based on 21 complete cp genome of Fagaceae and two *Corylus* species designated as outgroups. The bootstrap support values are shown above the node.

## Data Availability

The genome sequence data that support the findings of this study are openly available in GenBank of NCBI at [http://www.ncbi.nlm.nih.gov] (https://www.ncbi.nlm.nih.gov/) under the accession no. MW375417. The associated BioProject, SRA, and Biosample numbers are PRJNA684954, SRX9680931, and SAMN17073066, respectively.
